# Bovine Tuberculosis Prevalence Survey on Cattle in the Rural Livestock System of Torodi (Niger)

**DOI:** 10.1371/journal.pone.0024629

**Published:** 2011-09-22

**Authors:** Abdou Razac Boukary, Eric Thys, Emmanuel Abatih, Djibo Gamatié, Issoufou Ango, Alhassane Yenikoye, Claude Saegerman

**Affiliations:** 1 Department of Animal Health and Livestock Promotion, ONG Karkara, Niamey, Niger; 2 Department of Animal Health, Institute of Tropical Medicine, Antwerp, Belgium; 3 Faculty of Agronomy, University of Niamey, Niamey, Niger; 4 Department of Infectious and Parasitic Diseases, Research Unit of Epidemiology and Risk Analysis Applied to Veterinary Sciences (UREAR), Faculty of Veterinary Medicine, Université de Liège, Liège, Belgium; 5 Ministry of Agriculture and Livestock, Central Veterinary Laboratory, Niamey, Niger; 6 Ministry of Agriculture and Livestock, Direction of Animal Production, Niamey, Niger; St. Petersburg Pasteur Institute, Russian Federation

## Abstract

**Background:**

Bovine tuberculosis (BTB) is a widespread zoonosis in developing countries but has received little attention in sub-Saharan Africa, especially in Niger. Recent investigations confirmed the high incidence of the disease in cattle slaughtered in an abattoir in Niamey. The fact that most of the animals in which *M. bovis* has been identified were from the rural area of Torodi implied the existence of a probable source of BTB in this region. This study aimed to determine the prevalence of BTB infection in cattle and to identify risk factors for infection in human and cattle populations in Torodi.

**Methods and Principal Findings:**

A survey was carried out at the level of households keeping livestock (n = 51). The questionnaire was related to the potential risk factors and the presence of clinical signs of TB both in animals and humans. Comparative Intradermal Tuberculin Test was conducted to determine the TB status in cattle (n = 393). The overall apparent individual animal prevalence of tuberculin reactors was 3.6% (CI: 95%, 1.9–5.9), whereas the individual true prevalence was estimated at 0.8% (CI: 95%, 0.0–5.0). Using a multivariate logistic regression analysis and a classification tree analysis, the only household level risk factor that significantly influenced the presence of BTB in cattle was the presence of animals coughing in the herd (OR = 4.7, 95% CI: 1.12–19.71, p-value = 0.034). The lack of the practice of quarantine was borderline significant (OR = 4.2, 95% CI: 0.96–18.40, p-value = 0.056).

**Conclusion/Significance:**

The study confirmed that BTB is endemic in cattle in Torodi and the risk of the transmission of the disease to humans is potentially high. For the control of the disease in livestock, slaughtering of infected animals and the compensation of the owners is needed. Collaboration between the veterinary and the medical sectors, in the diagnosis, monitoring, prevention and control of BTB is strongly encouraged.

## Introduction

Tuberculosis due to *Mycobacterium bovis* is one of the seven most neglected endemic zoonoses in the world, particularly in developing countries [1]. In sub-Saharan Africa, bovine tuberculosis (BTB) causes significant economic losses. Indeed, the disease induces high animal morbidity and mortality that eventually reduces the financial capital and increases production costs [2], [3]. In addition, BTB represents a major threat to wildlife because it spreads rapidly, affecting a wide variety of animal species and likely to create a permanent reservoir of infection [4]. However, the relative importance of different sources of infection and routes of transmission in Africa are still largely unknown [5].

In Niger, opportunities exist for zoonotic transmission of *M. bovis*, including widespread consumption of unboiled milk, inadequate sanitation measures and extremely close human–livestock contact in rural as well as in urban communities [6]. However, little is known about the factors influencing the prevalence of bovine tuberculosis, or the principal risk factors for infection both in cattle and in humans. It should be noted that there is no formal program to control BTB in cattle in Niger [7]. Investigations carried out mainly on ranches and farms in Niger by single intradermal tuberculin test (SITT) showed that the disease occurred at relatively low prevalence levels ranging from 1.6 to 3.2% [8], [9]. In a follow-up study of 10 years (1974–1983), Alambedji [8] reported that on a total of 323, 339 cattle slaughtered at the abattoir of Niamey (capital of Niger), 2,538 were carrying TB-like lesions. Of these cattle with suspected BTB lesions, 43% came from the rural area of Torodi [8]. According to the author, this is linked to the intensive commercial livestock transactions between the capital city and its surrounding rural areas with Torodi district being the most important provider.

A recent study based on retrospective data analysis and a longitudinal survey in an abattoir in Niamey confirmed this trend. Boukary et al. [7] found that the rural zone of Torodi provided 56.2% of the observed BTB suspected gross lesions in the carcasses at the abattoir of Niamey. According to these authors, the prevalence of BTB-like lesions in cattle would be four times higher in Torodi than in other regions. The same authors isolated *M. bovis* from 18 animals and identified 4 different spoligotypes. Eleven of those 18 animals were from Torodi. Most *M. bovis* strains found in Niger such as SB1440, SB0944, SB0300 and SB1433 belong to type Af1 previously identified in Central and West Africa including Cameroon, Chad, Nigeria and Mali [10]–[14]. In addition, a profile (SB1982) not previously reported has been characterized in one Djeli cow from Torodi area [7]. So, the suspicion is high that cattle in the rural zone of Torodi may probably be an important source of BTB infection in Niger.

The current study builds on the aforementioned preliminary analysis and attempts to present, for the first time, more detailed results from an investigative study of risk factors for cattle and human *M. bovis* infection. The main objective of this project was to determine the prevalence of BTB infection in cattle in the rural area of Torodi.

## Materials and Methods

### Description of the study area and production system

**Figure 1:**
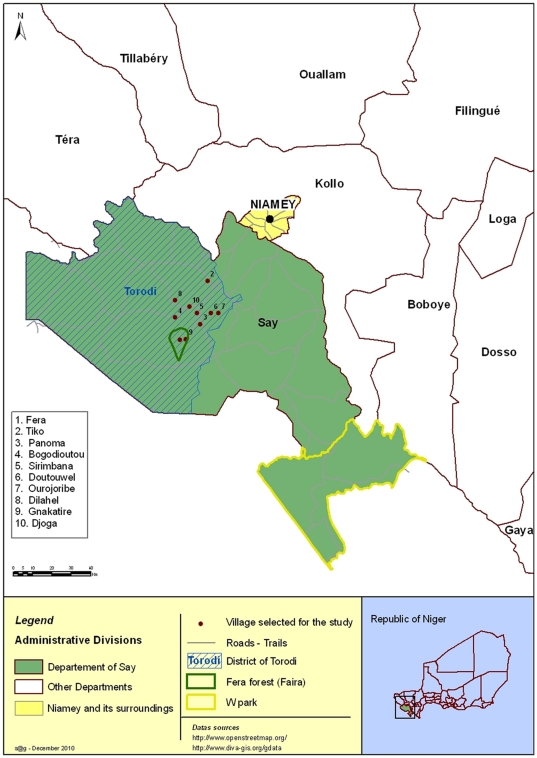
Location of the study zone (Torodi) in Niger.

The rural district of Torodi (**Figure 1**) is located in the Say department (Tillabery Region) in the far south-west of Niger at latitude 13°.7′9 N and longitude 1°.47′58 E. It covers 6978 km^2^, which corresponds to 62% of the total area of Say department. Torodi includes 105 administrative villages with an estimated population of 114,518 inhabitants [15]. Animals are managed under traditional husbandry system (extensive/transhumant). Livestock is mainly composed of cattle, sheep, goats and camel. They depend entirely on natural pastures and farm by-products without extra feed supplements and adequate health services. Cattle and small ruminants are usually driven to pasture together. Camels are generally led in separate herds and their feeding is mainly based on tree fodder. Livestock production is mainly oriented towards the production of slaughter animals. Milk is commonly used for home consumption. However, the excess milk is sold on rural markets.

Finally, because of its proximity to the W natural park stretched over Niger, Burkina Faso and Benin, Torodi is also an interface zone between domestic animals and wildlife (**Figure 1**). The W park is home to over 250 animal species. Of these, contact risk seems particularly high with herbivores such as elephant, buffalo, red hartebeest (*Alcelaphus buselaphus*), roan (*Hippotragus niger*) and bushbuck (*Tragelaphus scriptus*), but also with carnivores such as lion, cheetah, hyena, and leopard.

### Study design and data collection

The experiment took place between November 2007 and December 2009 in the rural zone of Torodi and was conducted in two phases. First, a cross-sectional household survey was carried out and the second step was the execution of the animal tuberculin test using comparative intra-dermal tuberculin testing (CITT).

#### The two stage cross-sectional household survey

It was carried out from November to December 2007. Firstly, ten villages were randomly sampled from the 105 rural villages of Torodi district. Secondly, 100 households keeping ruminants were proportionately and randomly extracted from up-to-date census databases of livestock keepers in the 10 villages (**Table 1**).

**Table 1:** Within herd apparent prevalence in local cattle (n = 393) in the rural zone of Torodi, Niger.

Herd (site)	Number of households interviewed per site	Number of household tested with CITT*	Number of animals tested	Number of positive animals	Number of households with positive animals	Apparent within herd prevalence (AP)	
						Average	95% CI:
Bogodioutou	6	3	8	0	0	-	-
Dilahel	11	3	53	0	0	-	-
Djoga	10	6	90	1	1	1.1	0.03–6.0
Doutouwel	10	4	21	0	0	-	-
Fera	6	2	6	0	0	-	-
Gnakatire	15	5	60	3	2	5.0	1.0–13.9
Ourojoribe	13	9	48	2	2	4.2	0.5–14.3
Panoma	9	5	23	2	2	8.7	1.1–28.0
Sirimbana	11	9	42	2	2	4.8	0.6–16.2
Tiko	9	5	42	4	4	9.5	2.7–22.6

Apparent prevalence (AP) and estimation of the true prevalence (TP) of bovine tuberculosis among local cattle (n = 393) in the rural zone of Torodi, Niger. TP was calculated using the value of Sensitivity and Specificity for the cut-off of >4 mm according to Ameni et al. (2008).

*Number of households in which animals were tested by comparative intra-dermal tuberculin testing (CITT).

The questionnaire used in the face-to-face interview with the head of the selected households included questions related to the social life of households, animal husbandry practices, food habits and the presence of clinical signs of TB both in animals and humans. After evaluation and screening of the results, 92 questionnaires were validated. The other 8 questionnaires included biases and were rejected for non-compliance with the selection criterion of keeping ruminants. Geographical coordinates were registered at the central point of each village by hand held global positioning system (GPS) instrument. For a better understanding of the relative importance and inter-relations between the various risk factors explaining the BTB status in cattle, data from both human and cattle surveys were compared. Out of the 92 households from the survey of 2007, only 51 were retrieved during the second investigation on BTB prevalence (see below). Only these 51 households were therefore included in the analysis on risk factors.

#### Determination of the apparent prevalence of BTB by comparative intra-dermal tuberculin testing (CITT)

Between September and December 2009, all cattle older than 4 years (393 heads) belonging to the 51 remaining households from the previous survey were tested with CITT. The choice of this class of animals was justified by the need to include all animals actually present in the herds at the moment of the previous investigation. In the study, cattle of each village are considered as one single epidemiological unit i.e. a group of animals for which the risk of transmission of a pathogen within the group is significantly higher than that between one animal in the group and an animal in another group.

The CITT was chosen because, compared to simple intra-dermal tuberculin testing (SITT) this technique has the advantage of differentiating between infections by *M. bovis* and those by other mycobacteria (including the avian group) [16], [17]. In addition, difficulties to conduct subsequent field investigations in case of preliminary use of SITT (recognized as a little more sensitive) were also taken into account. CITT was conducted as described in the OIE manual [17]. The operation is performed on the right side of the animal in the middle neck region. Two circular areas of about 2 cm^2^ diameter, about 12–15 cm apart, on the cervical area of the skin, were clipped with a curve scissors and disinfected with 70% ethanol. The initial skin thickness was measured with a calliper (Hauptner®). Respectively, 0.1 ml (20 000 IU/ml) of bovine tuberculin PPD (Bovituber PPD, Synbiotics Europe, Lyon, France) at one site and 0.1 ml (25 000 IU/ml) of avian tuberculin PPD produced in Lelystad (Netherlands) at the other site were injected into the dermis. Skin thickness was measured again at both injection sites after 72 hr. The reaction at each site was derived by measuring the difference of the skin thickness before and 72 hr after the injection.

The results were interpreted according to the recommendations of the World Organisation for Animal Health [17] using the cut-off point for positivity of the CITT test, calculated as the difference between skin thicknesses after bovine tuberculin (B) and avian tuberculin (A) injections (B – A). The test was considered positive if the difference was greater than 4 mm. The reaction was declared doubtful if the increase in skin thickness was between 2 and 4 mm.

#### Determination of the true prevalence of BTB

Because no data were available for Niger, specificity (Sp) and sensitivity (Se) for the CITT were estimated using the values of the study carried out in Ethiopia by Ameni et al. in 2008 [16]. The values of Se and Sp and their 95% confidence intervals at a cut-off of >4 mm based on this study were as follows: Se: 0.594 (95% CI: 0.489–0.693) and Sp: 0.969 (95% CI: 0.893–0.996).

The true prevalence (TP) was estimated by using the values of Sp and Se applied in the formula proposed by Rogan and Gladen [18]: TP = (AP+Sp-1)/(Se+Sp-1), where AP is the apparent prevalence; Se is the sensitivity and Sp is the specificity.

### Statistical analysis

#### Descriptive analysis

Descriptive statistics were used to compare the proportion of BTB infected households for different levels of the risk factors considered. All the risk factors considered for this study are listed in **Table 2**. Variables with more than 25% missing observations were not considered for analysis and only those deemed as relevant risk factors were examined. Categorical variables with more than two levels were coded as dummy variables. Logistic regression was employed to examine the effects of sex and age on BTB infection in cattle.

**Table 2:** Univariate analysis of raw data indicating potential risk factors associated with bovine tuberculosis (BTB) in cattle and humans in the rural area of Torodi, Niger.

Category	Variable Code	Level	Proportion	Odds ratio	95% CI	*P*-value
**Location**	Site	Name of the village (also designates the epidemiological unit = herd) where surveys were conducted: 1 Bogodioutou; Dilahel 2, 3 …, 10 Tiko.				
**Social characteristics of the household**	Age	Age of head of household (interviewee)		1.0	0.9–1.0	0.429
	Ethnic group	Ethnic group of head of household:				
		*1 : Fulani*	76.5	Ref	-	-
		*2 : Zarma*	19.6	0.6	0.1–3.4	0.602
		*3 : Gurma*	3.9	ND		
	Education	Educational level of head of household				
		*1: Any level*	87.8	Ref	-	-
		*2: Literate (can read and write only)*	6.1	0.9	0.7–12.1	0.955
		*3: Primary school level*	6.1	0.5	0.1–2.0	0.337
		*4: Educated (level of high school, university)*	0	0.9	0.3–3.1	0.920
	Household size	Number of people living in the household		1.0	0.9–1.1	0.633
	Marital status	Number of wives of the household head.				
		*1: monogamous (only 1 wife)*	66.7	Ref	-	-
		*2 : polygamous (2 or more than 2 wives)*	33.2	1.6	0.4–6.2	0.481
**Practices**	Husbandry	Importance of livestock in the socio-economic aspects of the household (occupation of working time and economic contribution)				
**related to livestock**		*1: High*	45.7	Ref	-	-
		*2: Moderate*	45.6	1.3	0.3–5.0	0.726
		*3: Low*	8.7	1.0	0.9–12.7	0.959
	Crops	Importance of crops in the socio-economy aspects of the household (occupation of working time and economic contribution)				
		*1: High*	60.9	Ref	-	-
		*2: Low*	39.1	1.4	0.4–5.6	0.623
	Herd size	Number of cattle owned by the household		1	0.9–1.0	0.588
	Handling*	Handling of newly arrival animals (mixed with others animals or quarantined)				
		*1: Mixed*	74.5	Ref	-	-
		*2: Quarantine*	25.5	3.8	1.0–14.9	0.055
	Milk consumption	Type of dairy product usually consumed within the household.				
		*1: Unpasteurized milk*	68.6	Ref	-	-
		*2: Pasteurized milk*	9.8	0.7	0.7–7.3	0.783
		*3: Milk powder*	21.6	1.0	0.2–5.0	0.918
	Hygiene	How (with what) are utensils used for milking and milk processing cleaned				
		*Plain water*	51.0	Ref	-	-
		*Hot water*	13.7	1.1	0.2–6.9	0.931
		*Detergent*	7.8	ND	-	-
		*Other products*	27.5	1.1	0.3–4.6	0.911
**Reporting of clinical**	Weight loss	Presence of animals showing a state of severe weight loss despite a good diet and deworming	69.4	1.7	0.4–7.2	0.494
**signs of TB**	Animals cough*	Presence of animals suffering from chronic cough in the herd	43.1	4.3	1.1–4.8	0.034
**in animals and humans by interviewees**	Dead cough*	Whether or not animals died of chronic cough in the herd	52.9	2,5	0.7–9.5	0,18
	Entourage cough*	Presence of people suffering from chronic cough in the close environment of the household	54.9	2.3	0.6–8.6	0.235
	Household cough*	Presence of people suffering from chronic cough in the household	19.6	4.1	1.0–17.8	0.057
**CITT**	CITT**	Having or not at least one animal testing positive by CITT within the household *(1 having at least one animal tested positive by CITT within the household; 0: not having animals tested positive by CITT within the household)*				

*These values had *P*-value<0.25 and were identified as potential risk factors for inclusion in the multivariable analysis; Ref: indicate that the level is taken as the reference to which others are compared; ND: not determined;

**Target variable for multivariate analysis and CART analysis.

#### Examination of risk factors for BTB transmission

The risk factors for BTB were determined based on a two stage modelling approach. Initially, a univariate analysis was performed to study the association between each household factor and the presence or absence of bovine tuberculosis in the household's herd. Presence is defined as a positive CITT result for at least one animal from the household's herd. This was conducted by fitting a logistic regression model between each predictor variable individually on the outcome variable. Following the univariate analysis, only factors with a p-value <0.25 were selected as potential factors to be included in the multivariable logistic regression model. The most appropriate model was selected using backward stepwise selection. The effects of confounding were investigated by observing the change in the estimated odds ratios of the variables that remain in the model once a non-significant variable was removed. When the removal of a variable led to a change of more than 25%, that variable was considered a confounder and was not removed from the model. All pairwise interactions between the variables in the final model were examined for significance. Goodness of fit was assessed using the Hosmer-Lemeshow goodness-of-fit test. All statistical analyses were performed using STATA, version 11, software (Stata Corp LP, College station, Texas) [19].

Using the same set of significant variables from the univariate analysis, we conducted a classification and regression tree (CART) analysis [20] in an attempt to better understand the relative importance and inter-relations among different risk factors in explaining BTB status in cattle. CART is a non-parametric method used for identifying predictor variables by using binary recursive partitioning. In this method subsets of households are formed by examining each possible cut point of each variable to identify the cut point that resulted in maximum discrimination between subgroups of households with respect to the probability of BTB infection.

## Results

### Apparent and true prevalence of BTB

The comparative intradermal tuberculin test (CITT) focused on 393 cattle (*Bos indicus*) of which 99% were of the Djeli breed, 2 (0.5%) were of the Azawak breed and 2 (0.5%) were crossbred. There were 36 bulls (9.2%) serving 357 cows (90.8%). The age distribution shows that 53.9% of the animals were between 4 and 7 years old, 29.0% between 8 and 10 years old and 17.0% were older than 10 years (**Table 3**). Of all animals tested, 23 (6.9%) presented doubtful results with CITT values between 2 and 4 mm. The results presented in **Table 1** show that the apparent (AP) and true (TP) prevalence of BTB was 5.6% (95% CI: 0.7–18.7) and 4.4% (CI: 95%, 0–27.6) in bulls respectively. In cows, the AP was 3.4% (CI: 95%, 1.7–5.8), while the estimated TP was 0.5% (CI: 95%, 0.0–4.8). There was no statistically significant difference between cows and bulls (Fisher's exact test; *P*>0.05).

**Table 3:** Apparent prevalence in local cattle (n = 393) in the rural zone of Torodi, Niger.

Age group	Sex	Number of animals tested	Number of positive animals	Apparent prevalence (AP) %		True prevalence (TP) %	
				AP	95% CI:	TP	95% CI:
4–7 years	Male	36	2	5.6%	0.7–18.7	4.4%	0–27.6
	Female	176	8	4.5%	2.0–8.8	2.6%	0–10.0
							
8–10 years	Male	0	0	-	-	-	-
	Female	114	1	0.9%	0.0–4.8	0.0%	0–3.0
							
>10 years	Male	0	0	-	-	-	-
	Female	67	3	4.5%	0.9–12.5	2.4%	0–16.8
							
	Total Male	36	2	5.6%	0.7–18.7	4.4%	0–27.6
	Total Female	357	12	3.4%	1.7–5.8	0.5%	0–4.8
**Total**	**N**	**393**	**14**	**3.6%**	**1.9–5.9**	**0.8%**	**0–5.0**

Apparent prevalence (AP) and estimation of the true prevalence (TP) of bovine tuberculosis among local cattle (n = 393) in the rural zone of Torodi, Niger. TP was calculated using the value of Sensitivity and Specificity for the cut-off of >4 mm according to [8].

The overall apparent individual animal prevalence of tuberculin reactors was 3.6% (CI: 95%, 1.9–5.9), whereas the individual true prevalence was estimated at 0.8% (CI: 95%, 0.0–5.0). The villages of Tiko and Panoma were the most affected with intra-herd apparent prevalence of 9.5% and of 8.7% respectively. No positive animals were detected in herds tested in the villages of Bogodioutou, Dilahel, Doutouwel and Fera. In addition, none of the assessed potential risk factors of BTB transmission in cattle such as age and sex were statistically significant.

### Risk factors for BTB transmission in humans and cattle

The results presented in **Table 2** show that households investigated in this study belonged mostly to the Fulani ethnic group (76.5%) while 19.6% were Zarma and 3.9% were from the Gurma ethnic group. The heads of interviewed households were mainly illiterates, since 87.8% of them had no grade level and none of them had exceeded the level of basic primary school. Husbandry occupies an important place in the household economy in Torodi area. The average number of cattle owned per household was 18 (±17) heads. About 60.9% of household heads surveyed considered that agriculture was the biggest business in terms of time occupation and contribution to household survival. Indeed, agricultural tasks are considered more difficult by the household members since they take up more time and manpower.

As concerns eating habits of the population, it should be noted that consumption of unpasteurized dairy products is a widespread practice. More than 68% of the households heads interviewed said that their families consumed exclusively unpasteurized milk, while 21.6% consumed powdered milk and 9.8% consumed pasteurized milk. Good hygienic practices were also lacking. Indeed, 51% of the surveyed households used only natural water (water from wells or creek water) to clean the equipment used for milking or processing of milk. The use of natural products such as leaves or tree bark was practiced by 27.5% of the households.

In terms of animal management, the practice of quarantine is well known to farmers though overall not commonly practiced. Eighty eight percent of respondents said they knew this technique but only 25.5% practiced quarantine. The duration of quarantine varies depending on the villages and livestock keeper. For those who practice in the region of Torodi, this period is 3 to 5 weeks (20 to 40 days) during which new animals are kept away from the rest of the herd. This period may allow farmers to observe the general body conditions of the animals and to detect possible clinical signs of diseases including symptoms associated with BTB. The presence of chronic cough (frequent cough sometimes accompanied by haemoptysis and nasal discharge for at least 3 months) appeared to be the main clinical symptom of TB both in animals and humans in the study area. Indeed, 43.1% of the interviewed household heads reported cases of chronic cough in animals and 53% acknowledged having had at least one case of death in animals showing this symptom within the last 5 years. Regarding human health, 55% and 19.6% of the respondents reported that they know people suffering from chronic cough in their immediate surroundings or in their own households respectively. Following the univariate analysis, 5 variables with p-values<0.25 (**Table 2**) were subjected to the multivariable analysis: the practice of quarantine (variable “Quarantine”), the presence of animals suffering from chronic cough in herds (variable “Animals cough”), the presence of dead animals due to chronic cough in herds (variable “Dead cough”), the presence of people with chronic cough in the entourage of the household (variable “Entourage cough”) and the presence of people suffering from chronic cough in the household (variable “Household cough”) (**Table 2**). The final multivariable model indicated that the only household level risk factor that significantly influenced the presence of bovine tuberculosis was the presence of animals coughing in the herd (*P*-value<0.05) (**Table 4**). The handling of newly arrived animals (quarantine) was identified as a confounder and was forced into the final model. However, there was no significant interaction between the two main effects. The Hosmer–Lemeshow test showed that the model fit the data well (χ^2^ = 0.00, df = 1, p-*value* = 0.95). It can be concluded from the results that households with the presence of cattle coughing had higher odds of BTB infection as compared to those where such animals were absent (OR 4.7, 95% CI: 1.12–19.71, p-value = 0.034). Similarly the odds of having BTB for those households where newly arriving animals were mixed with the other animals in the herd were 4 times higher than those where quarantine was practiced (OR 4.2, 95% CI: 0.96–18.40, p-value = 0.056).

**Table 4:** Results of the final multivariable logistic regression model using backward stepwise selection with significant household level risk factors for bovine tuberculosis in the Torodi zone of Niger.

Risk factor	Level	Odds ratio	95% CI	P-value
Animal cough	0	Ref	-	-
	1	4.7	1.1–19.7	0.034
Handling of newly arrivals (quarantine)	0*	Ref	-	-
	1**	4.2	1.0–18.4	0.056

Ref: indicate that the level is taken as the reference to which others are compared;

*: quarantine was practiced;

**: mixed newly arrivals animals.

The CART model retained the following variables as major determinants of the target variable “CITT” (presence of TB) in decreasing order of influence: animals cough, number of animals, age, handling, crops, household size, weight loss, entourage cough, hygiene and milk consumption (**Table 5**).

**Table 5:** Ranking of BTB risk factors by overall discriminatory power using CART.

Variable	Discriminatory power
Animals cough	100
Number of animals	99.2
Age	76.4
Handling (quarantine)	46.3
Crops	37.0
Household size	34.6
Weight loss	24.5
Entourage cough	16.9
Hygiene	0.9
Milk consumption	0.03

**Figure 2:**
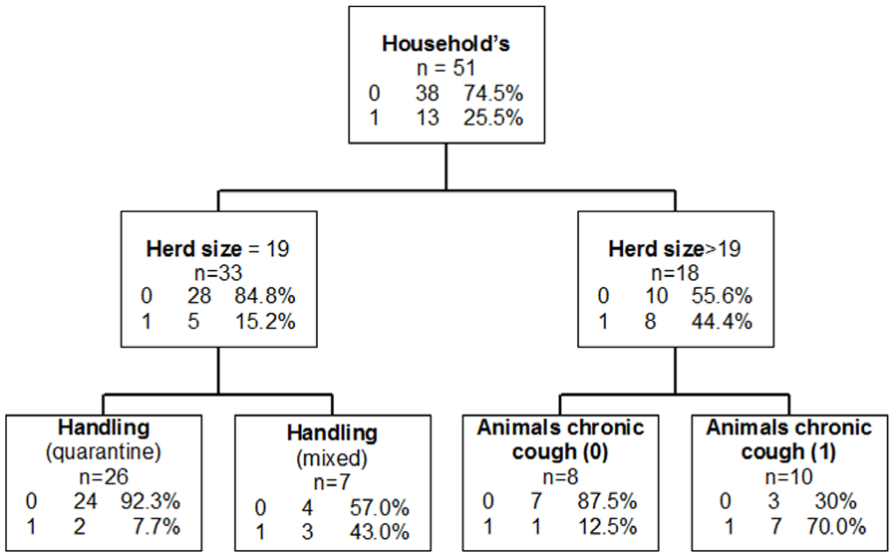
Classification tree produced using CART with target variable “CITT” (1 having at least one animal tested positive by CITT within the household; 0: not having animals tested positive by CITT within the household). Classification tree representing the important factors influencing CITT positivity. Target variable is “CITT” (1 having at least one animal tested positive by CITT within the household; 0: not having animals tested positive by CITT within the household). The following variables were selected by the tree as important factors: herd size with a cut off of 19 cattle in the herd; Handling (quarantine: keeping newly arrived animals out of the herd – mixed: new animals mixed with the other animals); Animals chronic cough (0 = absence of animals with chronic cough in the herd and 1 = presence of animals with chronic cough).

**Figure 2** shows the optimal classification tree as produced by CART. The 51 households in which animals were tested by CITT were initially divided into two intermediate nodes based on the number of animals. The classification tree ends in four terminal nodes: a terminal group of households mixing the new animals with the others (group “mixed”) (n = 7), a subgroup of heads of households who practice quarantine (n = 26), a subgroup of households where the animals never suffered from chronic cough (n = 8) and a subgroup of households with animals suffering from chronic cough (n = 10). The latter group was observed in 70.0% of households having animals reacting positively to CITT.

## Discussion

### Comparative intra-dermal tuberculin testing

Studies on the determination of the TP of BTB in West Africa are relatively rare. Also, regarding the AP of BTB, the results obtained by different authors using the intradermal tuberculin skin test are hardly comparable. Indeed, the performance of the tuberculin skin test can be affected by environmental factors, the prevalence of TB, host factors such as status of immunity, genetics, etc., and the nature of the tuberculin test used [21]. In addition, the cut-off point ideal for one group of cattle in a specific geographic area may be less ideal for another group in another environment [16]. Due to the paucity of local values for sensitivity (Se) and specificity (Sp) of the comparative intradermal tuberculin skin test (CITT), we estimated the TP of BTB using the results obtained by Ameni et al. [16] on cattle (*Bos indicus*) in the central region of Ethiopia. The values of Se and Sp found in Ethiopian local cattle breeds seemed more appropriate in the context of Niger compared to official values of the OIE [17] which were determined on European cattle (*Bos taurus*).

The low global TP of infection of cattle with *M. bovis* (0.8%) is in accordance with the results of Boukary et al. [7] who reported low rates of gross lesions suspected tuberculosis in cattle carcasses at the Niamey abattoir, where the average gross visible lesions was 0.19% (95% CI: 0.15–0.23) with a dominance of lesions in animals from Torodi. The low prevalence of BTB at the Niamey abattoir can be explained in part by the fact that the classical meat inspection is not able to detect all tuberculosis infected animals with confirmed lesions [22]. The TP of BTB in the rural area of Torodi is similar to that reported by Delafosse et al. [23] in the region of Abeche in Chad which was estimated to be 0.8%. The overall AP from our study (3.6%) is slightly higher than that reported by Alambedgi [8] and Bloch and Diallo [9] who found AP values between 1.6 and 3.2% using the single intradermal tuberculin test (SITT) on cattle reared in extensive systems in Niger. The prevalence of BTB is generally quite low in rural Africa where production systems are extensive. Cleaveland et al. [5] found an AP of 0.9% by CITT in cattle in the rural areas of Tanzania while Tschopp et al. [24] found an AP of 3% using the same test in rural areas in Ethiopia. On the contrary, our results regarding the AP of BTB in Torodi are lower than those reported by several authors in the urban and suburban African farms. For example, Cadmus et al. [25] found an AP of 10.5% by CITT in cattle in the city of Ibadan in Nigeria. Similarly Regassa et al. [26] found an individual AP of 11.6% in cattle through a study based on CITT conducted in Hawassa town and surroundings in Ethiopia. Sidibé et al. [27] and Traoré et al. [28] found an individual AP of 18.6% and 27.7% respectively by CITT and SITT in cattle raised on intra-urban and suburban systems in Bamako and Ouagadougou. Although the region of Torodi can be considered a potential source of BTB in Niger [7], [8], the individual prevalence of this disease remains low. This can be explained by the extensive nature of livestock keeping in the region. Indeed, the combination of adverse environmental conditions and extensive livestock practices could explain the low spread of BTB infection [3]. It should also be noted that our experiments were conducted during an extremely dry year and we noted in our study area, a strong seasonal movement of livestock to the natural reserve of W which is wetter and where animals are provided with feed.

### Risk factors for BTB transmission in human and cattle

#### Risk factors in cattle

Research conducted in the pastoral areas of Africa have shown that environmental factors such as sharing water points, grazing or high promiscuity are potential risk factors for transmission of BTB among animals [3], [29]–[31]. Our results showed that the presence of chronic cough in animals is closely linked to CITT reaction (OR = 4.7, 95% CI: 1.12–19.71, p-value = 0.034). The analysis performed with CART software has also shown that chronic cough in animals is the most important determinant of BTB infection in the context of Torodi. The predominance of suspect BTB gross lesions in the respiratory tract of cattle in previous studies conducted at the Niamey abattoir [7] has suggested that the respiratory route is the principal mode of transmission. Our results corroborate those of several authors who reported that aerosols are the main route of cattle-to-cattle transmission [32]–[34]. Similarly, several other authors [35], [36] found a significant association between respiratory pathology and reactivity to CITT. We found that the practice of quarantine reduced the presence of animals reacting positively to CITT within herds (OR 4.2, 95% CI: 0.96–18.40, p-value = 0.056). A possible reason for this is that as quarantine is not practiced, the risk of introducing infected animals into an infection free herd becomes higher [36]. It is obvious that the practice of quarantine would allow farmers to detect early clinical signs of BTB on new animals and would allow them to take appropriate health measures to protect the rest of the herd. In addition, the use of intra-dermal tuberculin testing upon arrival of new animals may drastically increase the chance to detect infected animals before their introduction into the herd. In accordance with findings from other studies [35], [37], our results show that, as herd size increased, there was a corresponding increase in the prevalence of BTB. Households with more than 19 heads of cattle were more likely to have animals infected with BTB. Hence, with increase in herd size, the risk of introducing an infected animal into a clean herd also becomes higher [5], [38]. The effect of age of cattle on the prevalence of BTB has also been reported. According to some authors, cattle older than 10 years are at higher risk of infection [5], [23]. It was also suggested that the duration of exposure increases with age with older cattle more likely to have been exposed than younger ones [2], [30], [39]. However, in our case, age was not statistically significant. This might have been due to the low number of older animals in our study (n = 67; 17%) probably caused by the fact that older animals showing clinical signs of BTB are removed from the herd and sold in the market of Torodi for slaughter. This proposal is supported by recent observations from other authors [7].

#### Risk factors in humans

According to Diguimbaye [40] and Humblet et al. [30], the proportion of human cases of tuberculosis due to *M. bovis* also depends on the importance of infection in animals within herds. Our results show a significant association between the presence of animals with chronic cough and the presence of cattle in the households reacting to CITT. This suggests that the disease can be transmitted to humans through the inhalation of cough sprays from infected cattle [36]. This assertion is further sustained by Ayele et al. [41] who noted that pulmonary TB due to *Mycobacterium bovis* is more common in rural dwellers as a result of inhalation of dust particles or bacteria-containing aerosols shed by infected animals. The fact that consumption of unpasteurized dairy products is a very common practice among farmers (68.6%) could not be statistically related to positive animal response to CITT. It should be noted however that the analysis using the CART software shows that hygiene in households and consumption of raw milk are among the determinants of *M. bovis* infection although with low discriminatory power in our case. It is well established that cattle infected with *M. bovis* can excrete the bacillus in their milk [2]. According to Cosivi et al. [3], cases of human TB of animal origin pose a serious public health problem, especially in areas where raw milk or its products are commonly consumed. Indeed, several researchers have shown that transmission of *M. bovis* from animals to humans is closely related to food habits and hygienic conditions of the population within households and the society in general [5], [27], [29].

### Conclusion

This study represents one of the first investigations on BTB prevalence and analysis of factors that promote the spread of the disease in the rural context of Torodi. The study was based on a sample design which is a major limitation in interpreting the results. Therefore, further investigations are needed to assess the extent of the disease. Nevertheless, the results achieved enable us to conclude that the risk of BTB transmission from animals to humans and humans to animals appear potentially high in view of animal husbandry and the eating habits of the population such as consumption of unpasteurized milk.

The main transmission route of the pathogen from animals to humans and vice-versa seems to be aerosol. Indeed, in cattle, chronic cough appears to be the main factor correlated with CITT following a multivariate analysis and based on the main discriminatory power in CART analysis. This is in accordance with the fact that most of suspected BTB lesions in animals were localized in the lungs [7]. The study underscores the need to test for BTB in all animals presenting chronic cough, also upon arrival of new animals and to identify local standards for determining the TP of BTB for livestock in Niger, particularly by determining a local cut-off value for CITT. The eradication of tuberculosis from livestock is expensive since it requires intensive surveillance of livestock, slaughtering of infected livestock, and the compensation of those who own each slaughtered, infected animal. The use and the appropriateness of the ‘test-and-slaughter’ strategy, which has already proved to be efficient in many industrialized countries, should be introduced in some endemic areas such as Torodi.

It is also important to investigate the epidemiological impact of BTB in humans through the determination of the TP and the detection of *M. bovis* in humans. In this context, coordinated actions involving researchers and institutions dealing with human and animal health are highly needed. Finally, as Torodi is an important crossway for cattle trade and transhumance and located at the edge of the W Natural Park, it is therefore important to implement additional studies to highlight the impact of the wildlife-domestic animal interface and transhumance on the transmission of the disease.
